# Integrative single-cell and spatial transcriptomics analysis reveals FLAD1 as a regulator of the immune microenvironment in hepatocellular carcinoma

**DOI:** 10.3389/fimmu.2025.1680101

**Published:** 2025-10-29

**Authors:** Peng Zhu, Lisha Mou, Ying Lu, Zuhui Pu, Changchun Guo

**Affiliations:** ^1^ Clinical Laboratory, Shenzhen Pingshan District People’s Hospital, Pingshan Hospital, Southern Medical University, Shenzhen, Guangdong, China; ^2^ Institute of Translational Medicine, The First Affiliated Hospital of Shenzhen University, Shenzhen Second People’s Hospital, Shenzhen, Guangdong, China; ^3^ MetaLife Lab, Shenzhen Institute of Translational Medicine, Shenzhen, China; ^4^ Imaging Department, The First Affiliated Hospital of Shenzhen University, Shenzhen Second People’s Hospital, Shenzhen, China; ^5^ Anesthesiology Department, Pingshan District Central Hospital of Shenzhen, Shenzhen, Guangdong, China

**Keywords:** hepatocellular carcinoma, mitochondrial function, single cell, spatial transcriptomics, machine learning, FLAD1, therapeutic targets, immune cell infiltration

## Abstract

Hepatocellular carcinoma (HCC) remains one of the leading causes of cancer-related mortality worldwide, characterized by increasing incidence rates and challenging prognoses. This study integrates single-cell RNA sequencing (scRNA-seq) and spatial transcriptomics to unravel the complex molecular and structural landscape of HCC, focusing on the identification of mitochondrial-related genes (MitRGs) and their pivotal role in disease progression. Utilizing scRNA-seq and bulk RNA-seq data, we performed a comprehensive differential expression analysis to highlight MitRGs. A modeling approach using 92 combinations of nine machine learning algorithms was applied, producing a predictive model with good performance. Among the genes analyzed, FLAD1 emerged as significantly upregulated in HCC tissues, correlating with advanced disease stages and poorer patient outcomes, and exhibited exceptional diagnostic accuracy with an AUC of 0.962. Functional enrichment analyses revealed that high FLAD1 expression is involved in crucial biological processes like copper ion detoxification and heme complex assembly. Interaction networks further elucidated the connection between FLAD1 and critical HCC pathways, with its expression levels negatively correlated with key immune effector cells such as CD8+ T cells and DCs. Spatial transcriptomics analysis provided a structural basis for this immune exclusion, demonstrating that an intact tumor capsule can function as a physical barrier that fosters an immune-exempt microenvironment. This analysis also validated FLAD1 upregulation within the spatial context of the tumor. Additionally, DNA methylation analysis indicated a hypomethylation pattern in the FLAD1 promoter region, likely contributing to its overexpression in HCC. Validation of FLAD1 protein levels in an in-house cohort via Western blotting further confirmed these findings. Collectively, our integrative study highlights the utility of MitRGs as potential biomarkers and positions FLAD1 as a dual prognostic and therapeutic target linked to the structural and immune landscape of HCC.

## Introduction

Hepatocellular carcinoma (HCC) is a prevalent primary liver cancer, posing a significant global health burden with high mortality rates (1). Despite advancements in diagnosis and treatment, the 5-year survival rate remains below 15%, largely due to late-stage presentation and limited treatment options (2). The incidence is highest in regions with endemic HBV, but rising trends are observed in Western countries due to HCV and metabolic syndrome (3). Current therapies, including surgery, transplantation, and systemic treatments, face challenges like tumor heterogeneity and drug resistance (4). Novel biomarkers and therapeutic targets are urgently needed to enhance HCC management.

Single-cell RNA sequencing (scRNA-seq) has emerged as a powerful tool to dissect the cellular complexity of HCC, revealing novel cell types and molecular pathways involved in tumor progression (5). Complementing this, spatial transcriptomics now allows for the mapping of these molecular profiles within the native tissue context, providing critical insights into the architectural features of the tumor microenvironment (TME) and its influence on processes like immune evasion (6, 7). Moreover, machine learning algorithms have shown promise in identifying gene signatures in HCC, which could serve as prognostic markers or therapeutic targets (8).

Flavin adenine nucleotide synthetase 1 (FLAD1) has been associated with mitochondrial functions. Research has revealed that FLAD1 is involved in the synthesis of flavin adenine dinucleotide (FAD), a crucial cofactor in mitochondrial energy production (9). Additionally, FLAD1 variants have been linked to multiple acyl-CoA dehydrogenase deficiencies, a condition affecting mitochondrial function (10). Interestingly, FLAD1 has been found to encode both cytoplasmic and mitochondrial transcripts, suggesting its role in mitochondrial processes (11). Overall, the involvement of FLAD1 in mitochondrial processes, particularly in FAD synthesis and mitochondrial disorders, underscores its significance in maintaining mitochondrial function and overall cellular health.

In cancer, FLAD1 has emerged as a promising therapeutic target. Studies have shown that FLAD1 is highly amplified in pancreatic cancer and is overexpressed in various cancers, correlating with poor prognosis (12–15). Coexpression analysis revealed that FLAD1 is coupregulated with other genes in HCC, suggesting potential interactions in cancer development (16). However, the role of FLAD1 in HCC is not well understood. Preliminary studies have indicated FLAD1’s involvement in tumorigenesis and cancer advancement, making it a promising HCC therapeutic target for further investigation.

This study integrates mRNA expression with clinical data from HCC patients to analyze scRNA-seq data, develop a machine-learning model for mitochondrial-related gene prediction, and conduct molecular experiments to validate the role of FLAD1 in HCC. We have identified distinct expression patterns of mitochondrial-related genes, developed a predictive model with high accuracy, and noted significant changes in FLAD1 expression. FLAD1’s involvement in immune cell infiltration and DNA methylation status underscores its potential as a diagnostic and therapeutic target in HCC. By employing scRNA-seq and machine learning, our study enhances the understanding of HCC’s molecular dynamics and sheds light on innovative targeted treatments.

## Methods

### Data collection

The bulk mRNA expression data and clinical information for hepatocellular carcinoma (HCC) patients were obtained from the following public databases:

1. TCGA-LIHC: Data in FPKM format were downloaded, including 50 paracancerous liver samples with mRNA data and 371 HCC samples with mRNA data. Among these, 341 HCC patients had both mRNA expression and clinical data (https://www.cancer.gov/tcga) (17).
2. ICGC-LIRI-JP: This dataset provided mRNA expression and clinical data for 212 HCC patients (http://lifeome.net:809/#/repository?type=buke) (18).
3. GTEx: A total of 110 normal liver samples were included as controls (https://xenabrowser.net/datapages/) (19).

To ensure consistency and standardized processing across all datasets, the data were uniformly processed using the Toil pipeline (20) from the UCSC XENA platform (https://xenabrowser.net/datapages/).

Single-cell RNA-seq data from ten patients with HCC were obtained from the GEO under accession number GSE149614 (21).

### Single-cell data analysis of HCC patients

This study analyzed single-cell data from three distinct HCC sample types. Group 1 consisted of primary tumor samples, while Group 2 comprised samples from portal vein tumor thrombus and metastatic lymph nodes. Data analysis was conducted using Seurat (v 4.1.0) (22), adhering to preprocessing thresholds from the original study (21): cells were filtered to include only those with mitochondrial gene content less than 10% and between 200 and 8000 RNA features. Normalization (NormalizeData), variable gene identification (FindVariableGenes), scaling (ScaleData), and principal component analysis (RunPCA) were performed using standard parameters. T-SNE (23) and RunHarmony (24) functions were applied to visualize data and adjust for batch effects, respectively. Cell type annotation utilized markers identified in the original study (21). Differential expression analysis was performed to identify genes that were significantly dysregulated in metastatic tissues (portal vein tumor thrombus and metastatic lymph nodes) compared to primary tumor tissues using the Wilcoxon method with an adjusted P-value cutoff of <0.05. Subsequently, differentially expressed mitochondrial-related genes (MitRGs) were identified among the DEGs, using the MitoCarta3.0 database as a reference (25).

### Spatial transcriptomics analysis of the tumor microenvironment

To investigate the spatial distribution of key MitRGs and the architectural features of the tumor microenvironment (TME), we performed a secondary analysis of public spatial transcriptomics data from a published study on primary liver cancer (GSA: HRA000437) (26). We focused on two representative paracancerous (L) sections to compare TME characteristics: sample HCC-4L, which is characterized by a complete fibrous capsule (defined as the CC group), and sample cHC-1L, which has a discontinuous or absent capsule (defined as the NC group).

The raw spatial transcriptomics data was preprocessed using Seurat (v4.4.0) (22). An automatic quality control process was applied to each sample to filter out low-quality spots based on the following criteria: (1) Fewer than 200 genes (nGene < 200) or fewer than 500 UMIs; (2) Mitochondrial gene content greater than 20%; (3) Spots located outside the main tissue area, as determined by H&E image calibration. For gene filtering, only genes expressed in at least three spots were retained for downstream analysis.

Data was normalized using the LogNormalize method in Seurat with a scale factor of 10,000. The top 2,000 highly variable genes were selected for subsequent analysis. To account for variability between samples, data from multiple slides were merged by patient source and corrected for batch effects using Harmony (v1.0) (24). The correction was performed by adjusting the top 20 principal components with patient source as the batch variable.

After data integration, unsupervised clustering was performed on the Harmony-corrected principal components using a shared nearest neighbor (SNN) graph algorithm. The resulting spatial clusters were visualized with UMAP (27) and annotated by combining H&E staining with differential gene analysis (Wilcoxon test with Bonferroni correction).

The abundance of 16 different cell types was quantified for each spot using the AddModuleScore function, which calculates a score based on the average log-normalized expression of marker gene sets. These cell types included various T-cell subtypes, M1/M2 macrophages, dendritic cells, and neutrophils. All scores were normalized within each cell type (from 0 to 1) for visualization and statistical comparison.

The expression of representative marker genes and immune checkpoint genes was visualized using spatial scatter plots, where color intensity represented expression levels. Group comparisons were conducted using the two-tailed Wilcoxon rank-sum test, with Bonferroni correction applied for multiple comparisons. Statistical significance was indicated as follows: *P < 0.05, ** P < 0.01, *** P < 0.001, and **** P < 0.0001. For visualizations, only spots with expression levels greater than zero were shown to minimize the effect of sparse data and highlight true expression differences. All analyses and visualizations were performed in R using packages including ggplot2 (v 3.5.1) (28) and ComplexHeatmap (2.20.0) (29).

### Establishment of a machine learning-driven predictive MITRG model for HCC patients

A predictive model for MitRGs in HCC was developed using nine machine learning algorithms with TCGA-LIHC cohort (341 HCC patients with both mRNA and clinical data) and validate with ICGC-LIRI-JP cohort (212 HCC patients with mRNA and with clinical data). Samples with missing survival data were excluded from the analysis. Machine learning algorithms include LASSO (30), Ridge (30), Elastic Net (Enet) (30), survival SVM (survivalSVM) (31), CoxBoost (32), Supervised Principal Components (SuperPC) (33, 34), Random Survival Forests (RSF) (35), Stepwise Cox (StepCox) (36), and partial least squares regression Cox (plsRcox) models (37). The performance of each model was assessed using AUC, ensuring a robust evaluation of predictive accuracy. The clinical endpoint for our prognostic model is overall survival (OS).

### Differential expression analysis of FLAD1

TCGA-LIHC cohort was divided into high and low FLAD1 expression groups based on the minimum P-value of FLAD1 expression. To explore differentially expressed genes (DEGs) between these groups, the R package DESeq2 was utilized (38). The criteria for DEGs were an adjusted P-value < 0.05 and an absolute log2-fold change (FC) > 1. Spearman correlation analysis was used to assess the correlation between the expression of the top 10 DEGs and FLAD1, which measures the strength and direction of the association between two variables.

### Western blotting analysis of in-house cohort

Sample collection in the study received ethical approval (Approval No.: 2025-333-05PJ and 2021-15). Key clinical characteristics of this cohort are summarized in **Supplementary Table S1**. Proteins were extracted using RIPA buffer (Beyotime, USA) enhanced with 1% PMSF. Subsequent separation of proteins was conducted via SDS-PAGE and the proteins were then transferred to PVDF membranes. These membranes were blocked with 5% nonfat milk and incubated overnight at 4 °C with primary antibodies against β-TUBULIN (#66240-1-Ig, Proteintech, Wuhan, China) and FLAD1 (#68491-1-Ig, Proteintech, Wuhan, China). Following primary antibody incubation, the membranes were exposed to the appropriate secondary antibodies. Detection of proteins was achieved through enhanced chemiluminescence. Semi-quantitative analysis of the proteins was performed using ImageJ software (v2.14.0) (39), allowing for measurement of protein levels.

### Analysis of FLAD1 protein expression from HPA database in HCC

The protein expression data of FLAD1 in HCC and normal liver tissues were derived from the Clinical Proteomic Tumor Analysis Consortium (CPTAC) via the Human Protein Atlas (HPA) database (http://www.proteinatlas.org/) (40, 41). The dataset comprised global protein abundance measurements obtained through mass spectrometry (MS) experiments utilizing isobaric tandem mass tags (TMT) for multiplexed quantification. Normalized relative protein expression (nRPX) values, calculated as log2(intensity), were directly obtained or calculated for analysis. This study included 165 HCC samples and 165 matched normal liver tissue samples.

### Function enrichment analysis

GSEA were used to investigate the biological functions associated with specific genes such as FLAD1. This tool, available through the MSigDB website (42, 43), aids in understanding the role of genes by employing GO and KEGG enrichment analyses using the R package clusterProfiler (v 4.4.4) (44, 45). Additionally, KEGG pathway analysis provided insights into the pathways that FLAD1 may influence, detailing the molecular interactions and signaling mechanisms involved. For reliable results, enrichment analyses adhere to strict thresholds: FDR < 0.05 and P < 0.05, ensuring the significance of the findings related to FLAD1.

### Interaction network analysis of FLAD1

The interaction network and related genes of FLAD1 were examined using GeneMANIA (46), a tool that identifies associations with input genes based on extensive datasets, including the interactions on protein and genetic.

### Analysis of immune cell infiltration

The extent of immune cell infiltration in HCC was evaluated using 24 distinct immune cells. The relative enrichment of these genes was quantified through a ssGSEA approach (47). We examined the association between FLAD1 and immune cells through Spearman correlation. To assess disparities in immune cell infiltration, we utilized the Wilcoxon rank-sum test to compare between the high and low FLAD1 expression groups, which provided insights into the immune landscape of HCC in relation to FLAD1 expression.

### DNA methylation analysis

For DNA methylation analysis, we utilized the UALCAN database (48) to assess the methylation status of the FLAD1 promoter, a key regulator of gene expression, and performed multivariate survival analysis using the MethSurv database (49). By correlating clinical outcomes with methylation data, our goal was to assess whether FLAD1 methylation status could function as a prognostic marker in HCC.

### Construction and validation of the nomogram

To forecast OS likelihood, we performed multivariable Cox regression analysis by R package survival (v 3.3.1) (50). We assessed the accuracy of this model using a calibration chart to compare the observed and predicted survival probabilities. Specifically, we employed a ​​bootstrap resampling method​​ with ​​200 repetitions​​ and a ​​sample size of 80​​ for each repetition to internally validate the model and mitigate overfitting. The calibration of the nomogram was evaluated using a ​​calibration curve​​, which graphically compares the predicted survival probabilities against the actual observed outcomes (as determined by the Kaplan-Meier method) at a specific time point (1, 3, or 5 years). All visualizations, including column and calibration charts, were generated using the R package rms (v 6.3-0) (51). These tools facilitated a clearer understanding of how prognostic factors influence survival outcomes, validating the model’s reliability and clinical applicability.

### Survival analysis

We analyzed survival data utilizing the Kaplan-Meier technique and log-rank test. The optimal cut-point for stratifying patients into high- and low-FLAD1 expression groups was determined using a maximally selected rank statistics method, which identifies the threshold that provides the most significant split in survival outcomes as determined by the log-rank test, using the survminer package (v 0.4.9) (52) and ggplot2 (v 3.5.1) (28). To assess the influence of clinical factors on HCC prognosis, univariate and multivariate Cox regression were performed. Variables showing significance at p < 0.1 in the univariate analysis were incorporated into the multivariate analysis.

### Protein structure prediction and molecular docking by AlphaFlod2 and AutoDock

We employed AlphaFold 2 (53), celebrated for its accuracy, rivaling direct observation methods such as cryo-electron microscopy, to predict the structures of FLAD1, a protein of particular interest in HCC. The amino acid sequence of FLAD1 was meticulously retrieved from the NCBI database (54). For molecular docking, AutoDock Vina 1.2.2 (http://autodock.scripps.edu/) (55, 56) was used, a proven software for analyzing the interactions and binding affinities between drug candidates and their protein targets. The molecular structures of Diethylstilbestrol, Thioacetamide, and Tretinoin were obtained from the PubChem database (https://www.ncbi.nlm.nih.gov/protein) (57).

Utilizing the predictive power of AlphaFold 2, we produced three-dimensional coordinates for FLAD1. The preparation for docking included converting all protein and ligand files to PDBQT format, involving the removal of water molecules and the addition of polar hydrogen atoms to ensure precision in the simulations. The docking grid box was strategically positioned to encompass the active domain of the target protein, facilitating optimal molecular interaction during the simulation. The dimensions of the grid box were set at 30 Å × 30 Å × 30 Å, with a grid point spacing of 0.05 nm, to efficiently capture detailed interaction data.

### Statistical analysis

We conducted our analyses using R (v 4.3.1) (58). A P < 0.05 was considered statistical significance, ensuring the rigor of our statistical conclusions.

## Results

### Single-cell RNA-seq analysis of hepatocellular carcinoma

**Figure 1:**
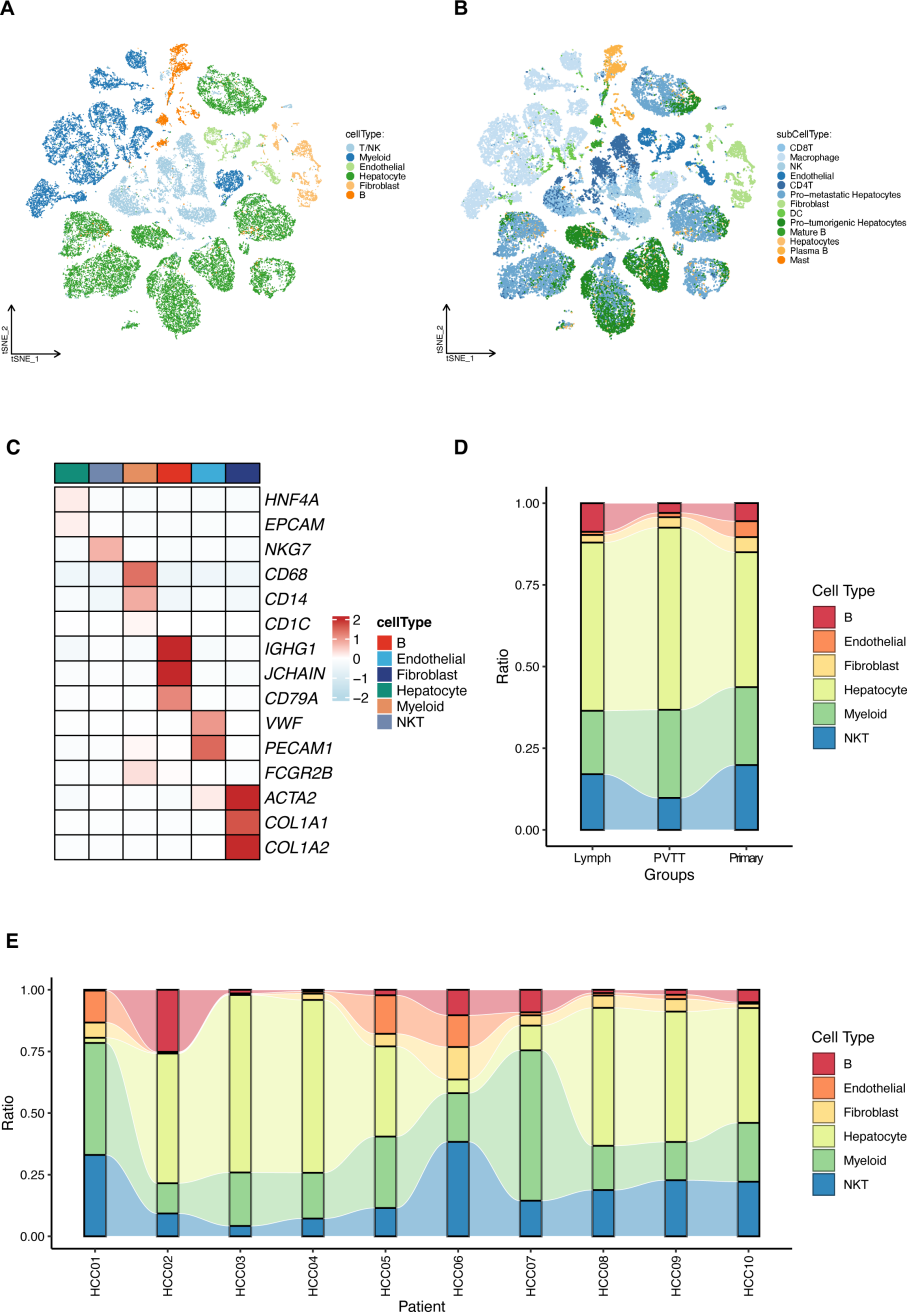
Comprehensive single-cell RNA sequencing analysis of hepatocellular carcinoma (HCC). **(A)** T-SNE visualization of the transcriptional landscape identifying six major types within primary and metastatic HCC. **(B)** Further resolution of the T-SNE plot showing 13 sub cell types across the sampled HCC conditions. **(C)** Detailed expression profiles of specific cellular markers across the six identified major cell types, highlighting their distinct molecular signatures. **(D)** Relative proportions of the cell type distributions in primary HCC tumors (Primary), portal vein tumor thrombus (PVTT), and metastatic lymph node samples (Lymph). **(E)** Relative proportions of the identified cell types across different HCC patients, illustrating inter-individual variability.

Single-cell RNA sequencing data (GSE149614) from the GEO database were analyzed for ten individuals with primary or metastatic hepatocellular carcinoma (HCC) (21). The initial cell clustering and annotation analysis serves as a replication and validation of the original study’s findings, performed to ensure consistency in data handling. We then clearly transition to our novel analysis focusing on the differential expression of mitochondrial-related genes. After stringent quality control as per the original study protocols, a total of 71,915 cell samples were processed. The data were normalized and dimensionally reduced before clustering into cell populations via t-SNE (**Figures 1A, B**). This analysis delineated six predominant cell types (hepatocytes, myeloid cells, T/NK cells, B cells, fibroblast cells, and endothelial cells) and 13 sub cell types across primary and metastatic HCC samples with the same cell markers as the original study (**Figure 1C**), with their distribution quantified in subsequent figures (**Figures 1D, E**).

**Figure 2:**
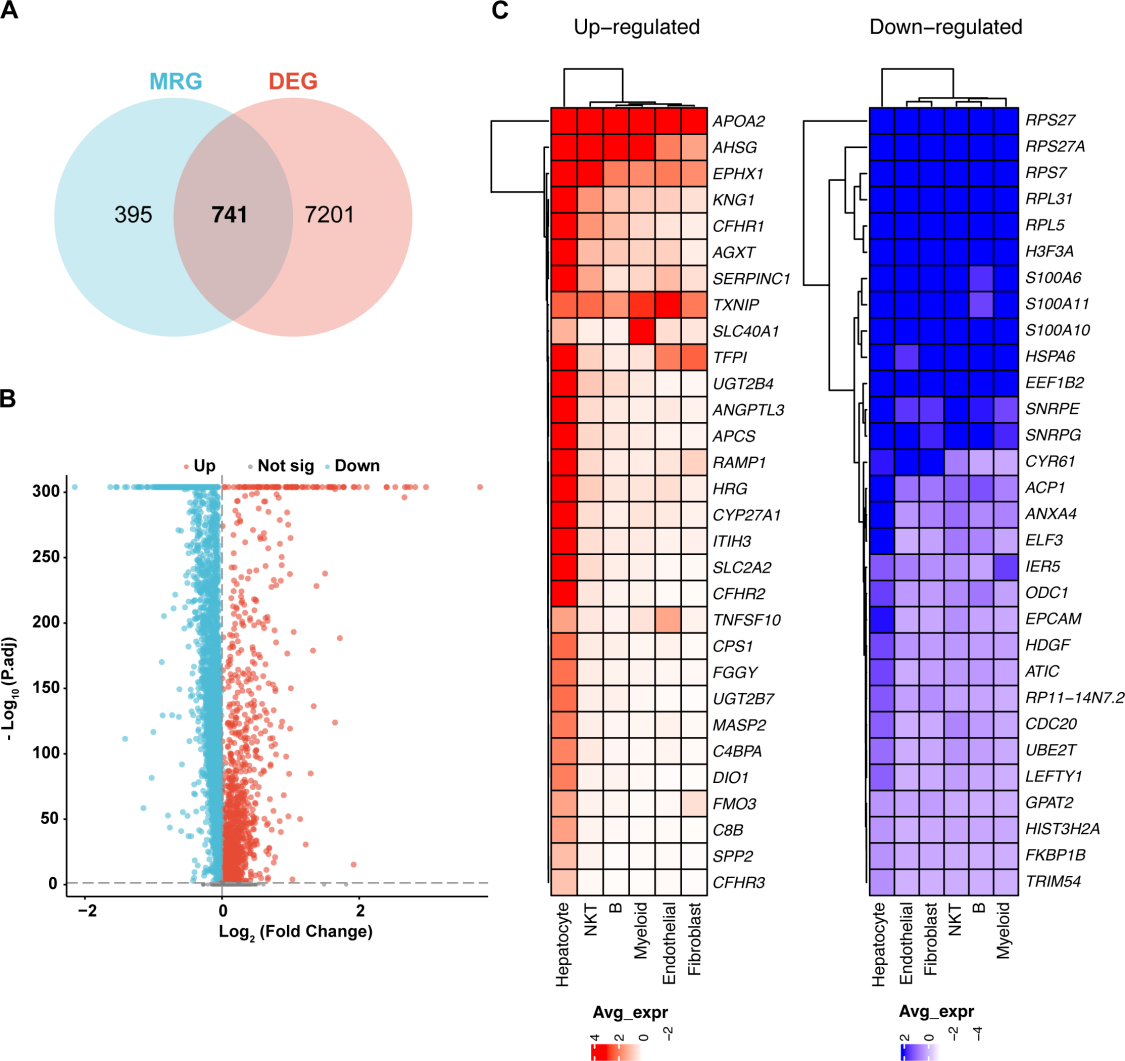
Differential expression of mitochondrial genes in HCC. **(A)** Discovery of 741 mitochondrial-related genes (MitRGs) that intersect with differentially expressed genes (DEG) in HCC, derived from an analysis incorporating MitoCarta3.0 data. **(B)** Volcano plot depicting significant upregulated and downregulated genes in metastatic versus primary HCC samples, emphasizing gene expression variations. **(C)** Heatmap presentation of the most significantly altered genes in metastatic HCC, comparing these changes to primary tumor profiles.

Differentially expressed genes (DEGs) were identified by comparing metastatic samples (including portal vein tumor thrombus and metastatic lymph nodes) to primary tumor samples. Key mitochondrial-related genes (MitRGs) were extracted from DEGs (**Supplementary Table S2**), with 741 MitRGs showing significant variation (**Figure 2A**). Of these, 200 were upregulated and 541 downregulated, as detailed in the volcano plots (**Figure 2B**). A heatmap further illustrated these expression patterns, underscoring the pivotal MitRGs in liver HCC (**Figure 2C**).

### Development of 92 predictive MitRG models using nine machine learning algorithms for HCC

**Figure 3:**
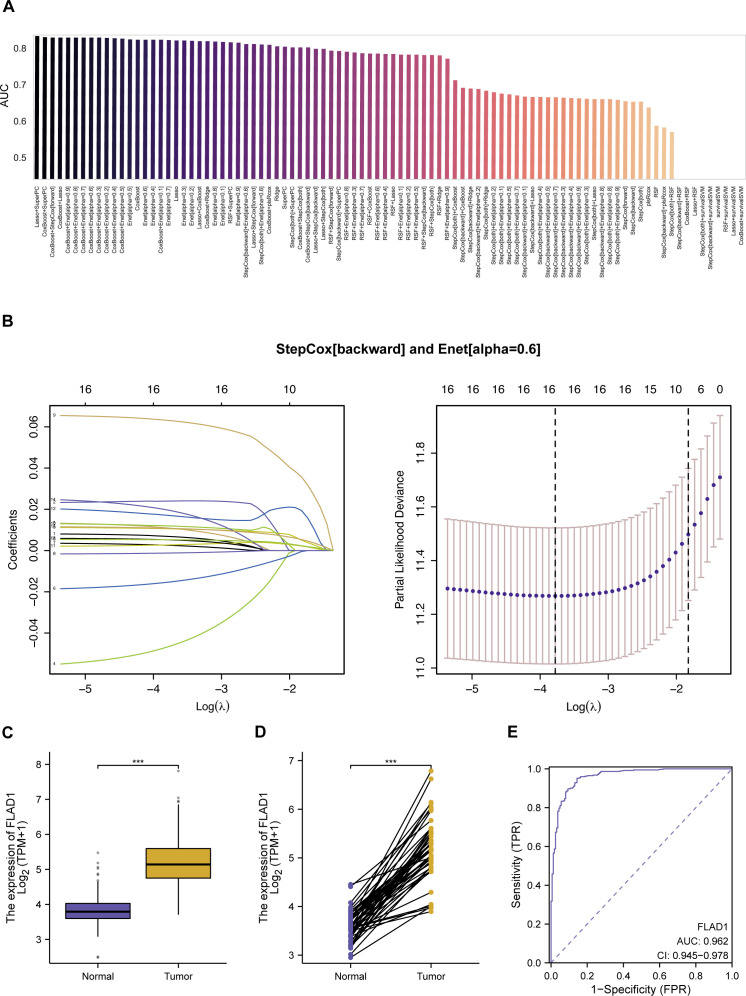
Development and evaluation of machine learning models for predicting mitochondrial gene expression in HCC. **(A)** AUC scores for 92 machine learning algorithm combinations, tested within TCGA cohorts, demonstrating model performance. **(B)** StepCox[backward] and Enet analysis. **(C)** The mRNA levels of FLAD1 in normal and HCC tissues, comparing data from TCGA and GTEx databases to showcase expression alterations. **(D)** Comparative analysis of FLAD1 expression in HCC tumors versus adjacent non-tumor liver tissues from TCGA, highlighting the genes with significant differential expression. **(E)** Receiver operating characteristic (ROC) curve demonstrates the diagnostic potential of the hub MitRG FLAD1 in HCC, with an AUC of 0.962, underscoring its high accuracy. ***P < 0.001.

We developed a predictive model employing a subset of differentially expressed MitRGs aimed at enhancing predictions of HCC progression. This model utilized nine advanced machine learning algorithms to construct and evaluate various predictive frameworks (**Figure 3A**). A rigorous validation process across 92 models identified an optimal combination using Lasso+SuperPC, which achieved an area under the curve (AUC) of 0.836 and identified 19 critical MitRGs. Another prominent model, illustrated in **Figure 3B**, combined StepCox[backward] and Enet[alpha=0.6]. This model focused on a specific set of 12 genes (ACACA, CLPX, DTYMK, ECI1, FLAD1, GRHPR, HTRA2, PAICS, PRDX6, SPTLC2, TXNRD1, UQCRH) and reached an AUC of 0.814 in the TCGA cohort. External validation was further conducted using independent ICGC-LIRI-JP datasets. The models demonstrated robust predictive performance in these cohorts, with 42 out of the 92 models achieving an AUC greater than 0.7 (**Supplementary Figure S1A**).

While the Lasso+SuperPC model did achieve the highest AUC of 0.836, we chose the StepCox[backward]+Enet[alpha=0.6] model (AUC = 0.814) for downstream analysis based on three key considerations: (1) Model Parsimony and Interpretability: The chosen model identified a more concise set of 12 genes, compared to 19 in the top-performing model. A smaller gene signature is more readily interpretable and has greater potential for translation into a cost-effective and efficient clinical diagnostic panel. (2) Performance Robustness: The difference in AUC (0.022) is marginal and may not represent a meaningful improvement in predictive power, especially when considering the risk of overfitting with more complex models. We argue that a slightly less performant but more parsimonious model may exhibit better generalizability. (3) Biological Coherence: The 12 genes in the selected signature (e.g., FLAD1, TXNRD1, UQCRH) have well-documented roles in mitochondrial metabolism and redox homeostasis, providing a stronger biological foundation for our subsequent, in-depth investigations into FLAD1. In summary, we believe the 12-gene model represents an optimal balance between achieving high predictive accuracy and ensuring model interpretability, clinical translatability, and biological relevance.

### Evaluation of mitochondrial-related genes as biomarkers in HCC

In the development of a predictive model utilizing a combination of StepCox[backward] and Elastic Net (Enet[alpha=0.6]), 12 genes were initially selected for detailed examination. Among these, 10 hub MitRGs (ACACA, DTYMK, ECI1, FLAD1, HTRA2, PAICS, PRDX6, SPTLC2, TXNRD1, and UQCRH) demonstrated significant upregulation in HCC tissues compared to healthy liver samples (**Supplementary Figure S2A**, **Supplementary Table S3**). These genes were were considered pivotal and retained for further analysis. Conversely, CLPX and GRHPR did not show significant upregulation in HCC tissues and were subsequently excluded from additional studies (**Supplementary Figure S2A**, **Supplementary Table S3**).

Further investigations revealed that the expression levels of these 10 hub MitRGs were considerably elevated in HCC tissues relative to paired adjacent non-tumor tissues in a cohort of 50 HCC patients (**Supplementary Figure S2B**, **Supplementary Table S4**). ROC curve analysis of 10 hub MitRGs were showed in **Supplementary Figure S2C**. One of the genes, FLAD1 showed upregulated expression in both HCC tissues compared to healthy liver samples (**Figure 3C**) and in HCC tissues relative to paired adjacent non-tumor tissues (**Figure 3D**). ROC curve analysis of FLAD1 specifically underscored its high diagnostic precision for HCC, achieving an AUC of 0.962 (**Figure 3E**). This substantiates the potential of FLAD1 as a robust biomarker for the detection of HCC.

### Prognostic value of FLAD1 in HCC

**Figure 4:**
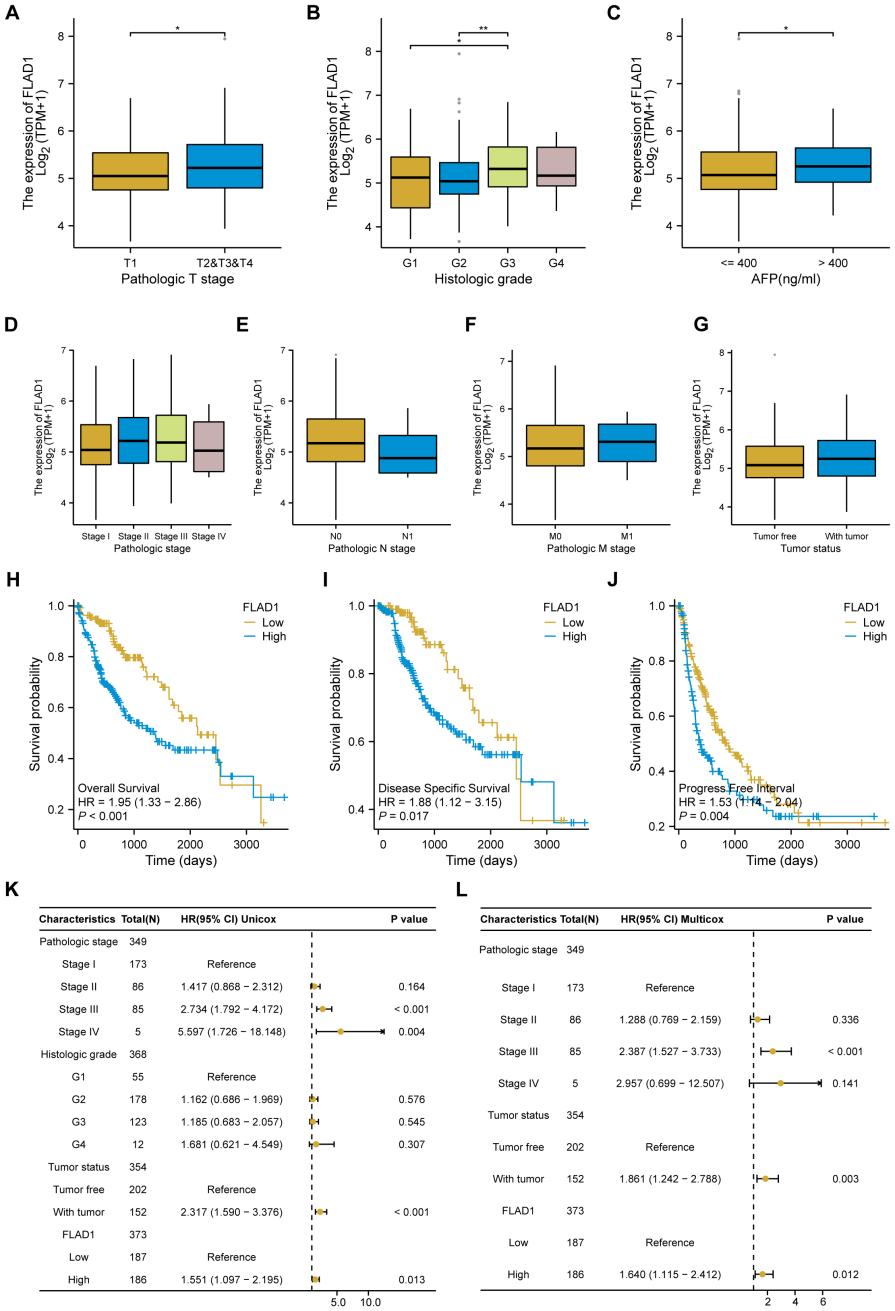
Associations between FLAD1 expression and clinicopathological features in patients with HCC, and Influence of FLAD1 levels on HCC prognosis assessed by Kaplan-Meier analysis. Correlations with **(A)** pathological T stage, **(B)** histological grade, **(C)** AFP level, **(D)** pathological stage, **(E)** N stage, **(F)** M stage, and **(G)** tumor status are presented, with significance denoted as *P < 0.05, **P < 0.01. **(H)** Overall survival (OS), **(I)** disease-specific survival (DSS), and **(J)** progression-free interval (PFI) between the high and low FLAD1 expression groups. Forest plot illustrating the impact of FLAD1 on OS based on **(K)** univariate and **(L)** multivariate Cox regression analyses.

Elevated FLAD1 expression was significantly correlated with more advanced pathological stages, particularly stages T2, T3, and T4, than with T1 (**Figure 4A**); grade G3, compared to G1; and grade G3, compared to G2 (**Figure 4B**). Additionally, FLAD1 levels were greater in the AFP>400 ng/ml group than in the AFP ≤ 400 ng/ml group (**Figure 4C**). However, no significant relationships were detected between FLAD1 expression and pathological stage, N stage, M stage, or tumor status (**Figures 4D–G**).

We employed the Kaplan-Meier method to investigate the prognostic implications of FLAD1 expression levels in HCC patients. Patients were stratified into high-FLAD1 and low-FLAD1 expression groups using the cutoff determined by the minimum P-value of FLAD1 expression. Notably, individuals in the high-FLAD1 expression group showed significantly poorer OS (**Figure 4H**), DSS (**Figures 4I**), and PFI (**Figures 4J**) compared to those in the low-FLAD1 group. A comprehensive analysis of prognostic factors in HCC was conducted using both univariate and multivariate Cox regression analyses. Key clinical characteristics, including FLAD1 expression, stage III disease, and tumor status, were identified as independent predictors of OS in HCC patients (**Figures 4K, L**).

**Figure 5:**
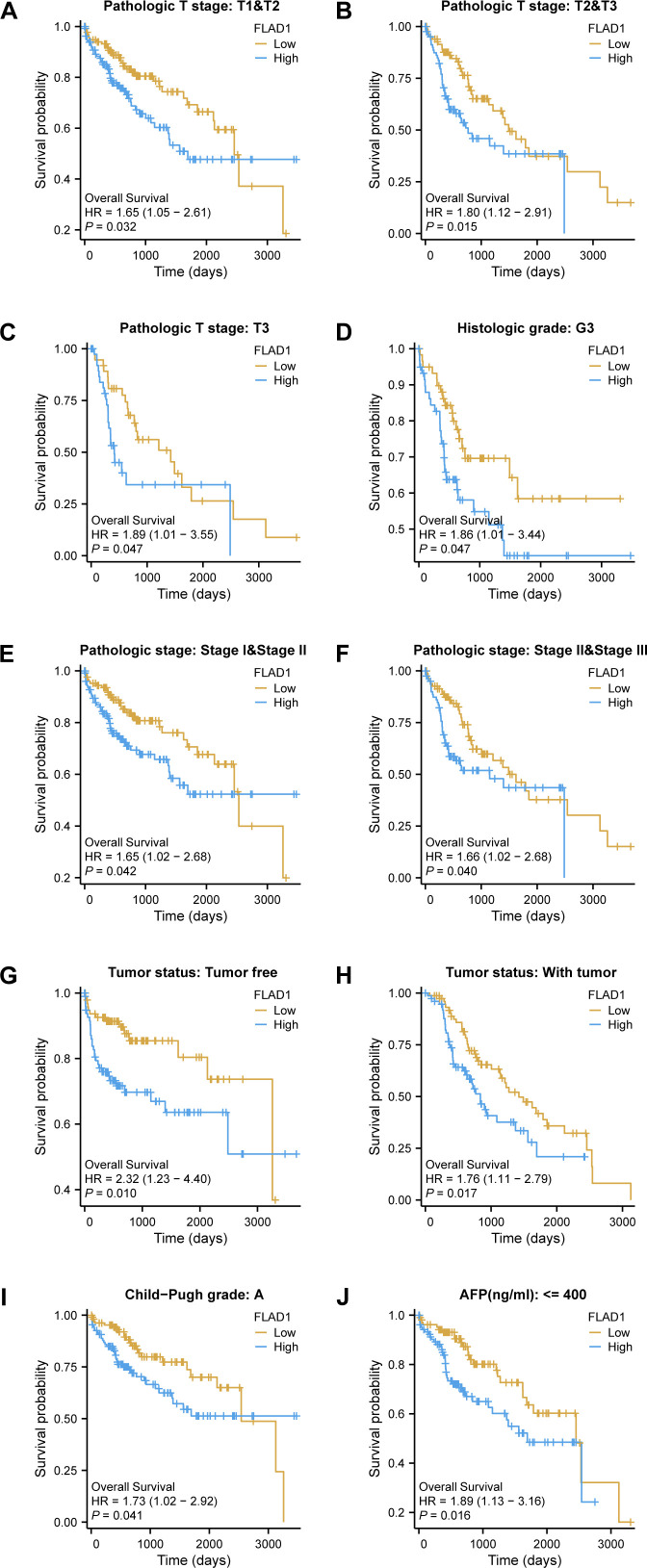
Kaplan-Meier analysis of the prognostic impact of FLAD1 levels across various subgroups of patients with HCC. Survival curves for **(A–J)**, including T1 *vs*. T2, T2 *vs*. T3, T3 alone, G3, Stage I *vs*. II, Stage II *vs*. III, tumor-free *vs*. with tumor, Child-Pugh grade A, and AFP levels below 400, comparing high and low FLAD1 expression groups.

Moreover, the prognostic significance of high FLAD1 expression was specifically analyzed across various subgroups. Significant disparities in prognosis were observed among patients in different stages or grades, particularly those in pathologic T stages, including T1 and T2, T2 and T3, T3, histologic grade G3, pathologic stages I and II, and stages II and III, and among patients who were tumor-free or had tumors, as well as those in Child-Pugh grade A and had AFP levels less than 400 ng/ml (**Figures 5A–J**).

### Functional enrichment analysis

**Figure 6:**
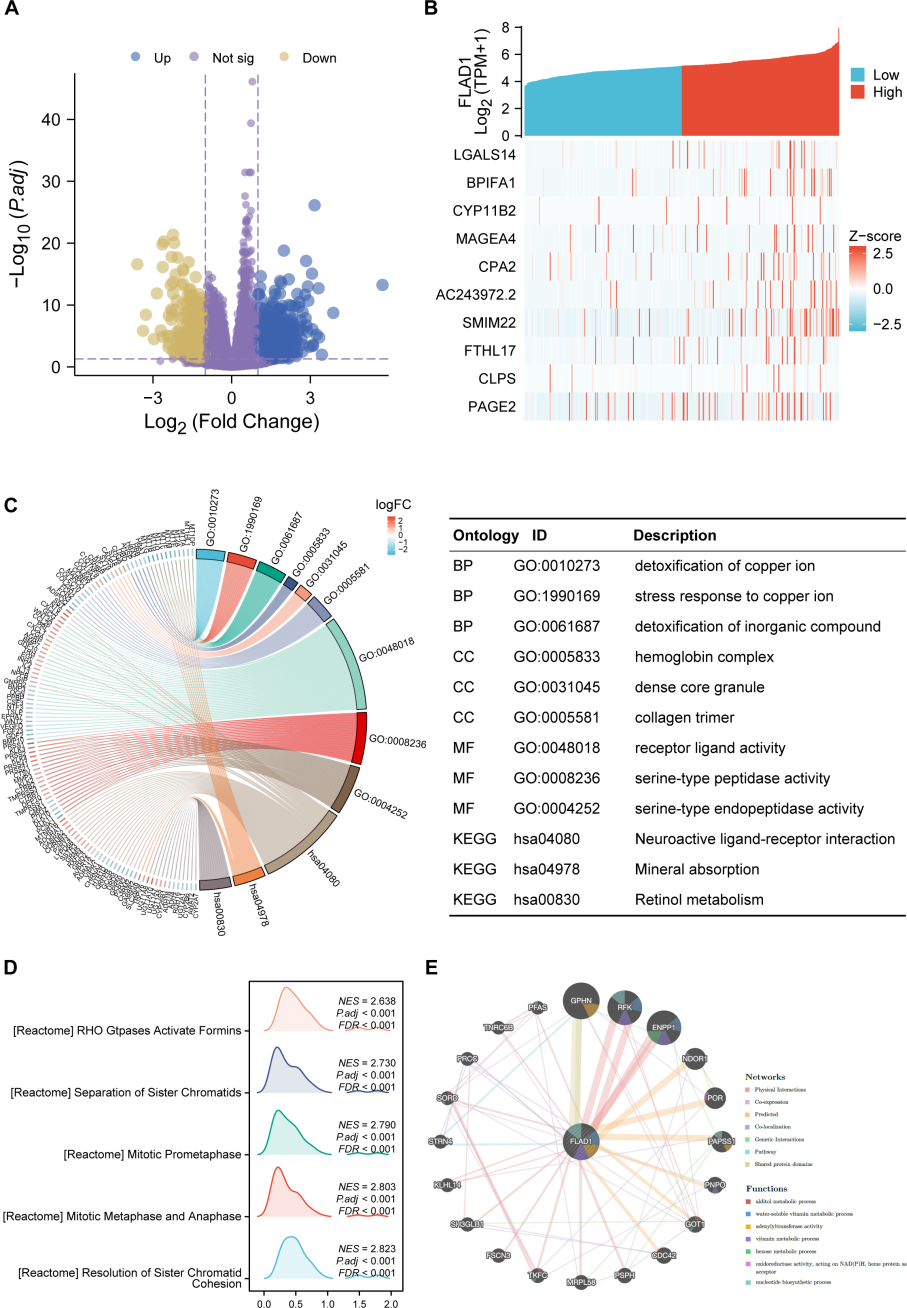
DEGs related to FLAD1 and their functional enrichment analysis. **(A)** Volcano plot showing upregulated (blue dots) and downregulated (yellow dots) DEGs between HCC tissues and paired cancer-adjacent tissues from TCGA data. **(B)** The top ten DEGs positively correlated with FLAD1. **(C)** GO and KEGG analyses of DEGs. **(D)** GSEA of DEGs. **(E)** GeneMANIA analysis showing the interaction network of FLAD1 and related genes.

First, we stratified the TCGA cohort based on FLAD1 expression to identify the global transcriptomic changes associated with FLAD1 levels (the DEGs). Analysis of DEGs showed that 1,131 coding DEGs between the high-FLAD1 and low-FLAD1 groups, with 692 genes up-regulated (61.18%) and 439 down-regulated (38.82%), adhering to adjusted P < 0.05 and |Log2 fold change| > 1 (**Figure 6A**; **Supplementary Table S5**).Subsequently, the correlation analysis between FLAD1 and the top 10 DEGs (**Figure 6B**; **Supplementary Table S6**) was performed to specifically illustrate the strength and direction of the linear relationship between FLAD1 and these key downstream effectors. This analysis serves to confirm that these genes are not just differentially expressed between the groups but are also tightly co-regulated with FLAD1 expression levels, providing more direct clues about the molecular network FLAD1 may influence.

GO and KEGG enrichment were conducted for all DEGs. The outcomes revealed that biological processes were predominantly enriched in response to the detoxification of copper ion, stress response to copper ion, and detoxification of inorganic compounds. Major cellular component enrichment included the hemoglobin complex, dense core granule, and collagen trimer. The enriched molecular functions were receptor ligand activity, serine-type peptidase activity, and serine-type endopeptidase activity. Notably, the most significant KEGG pathways included neuroactive ligand-receptor interaction, mineral absorption, and retinol metabolism (**Figure 6C**; **Supplementary Table S7**). Furthermore, GSEA revealed significant enrichment of processes such as the resolution of sister chromatid cohesion; stages of mitosis, including metaphase, anaphase, and prometaphase; and Rho GTPases activating formins in the high FLAD1 expression group (**Figure 6D**; **Supplementary Table S8**).

### Interaction networks of FLAD1

GeneMANIA analysis was utilized to construct a gene interaction network for FLAD1 (**Figure 6E**). Within this network, 20 genes functionally associated with FLAD1 were identified, including GPHN, RFK, ENPP1, NDOR1, POR, PAPSS1, PNPO, GOT1, CDC42, PSPH, MRPL58, TKFC, FSCN3, SH3GLB1, KLHL14, STRN4, SORD, PRCC, TNRC6B, and PFAS. To assess the clinical relevance of this network, we performed an overlap analysis between these 20 genes and the 1131 DEGs identified in our cohort. Significantly, we found that all 20 genes from the GeneMANIA functional network were also present in our list of DEGs. This finding confirms that these functionally linked genes are also transcriptionally dysregulated in HCC tumors with high FLAD1 expression. GeneMANIA pathway analysis connected these genes to FLAD1 through genetic, physical, or pathway links, highlighting seven significant pathways: alditol metabolic process, water-soluble vitamin metabolic process, adenylyltransferase activity, vitamin metabolic process, hexose metabolic process, oxidoreductase activity acting on NAD(P)H with heme protein as acceptor, and nucleotide biosynthetic process (**Figure 6E**).

### The association between FLAD1 expression and methylation

**Figure 7:**
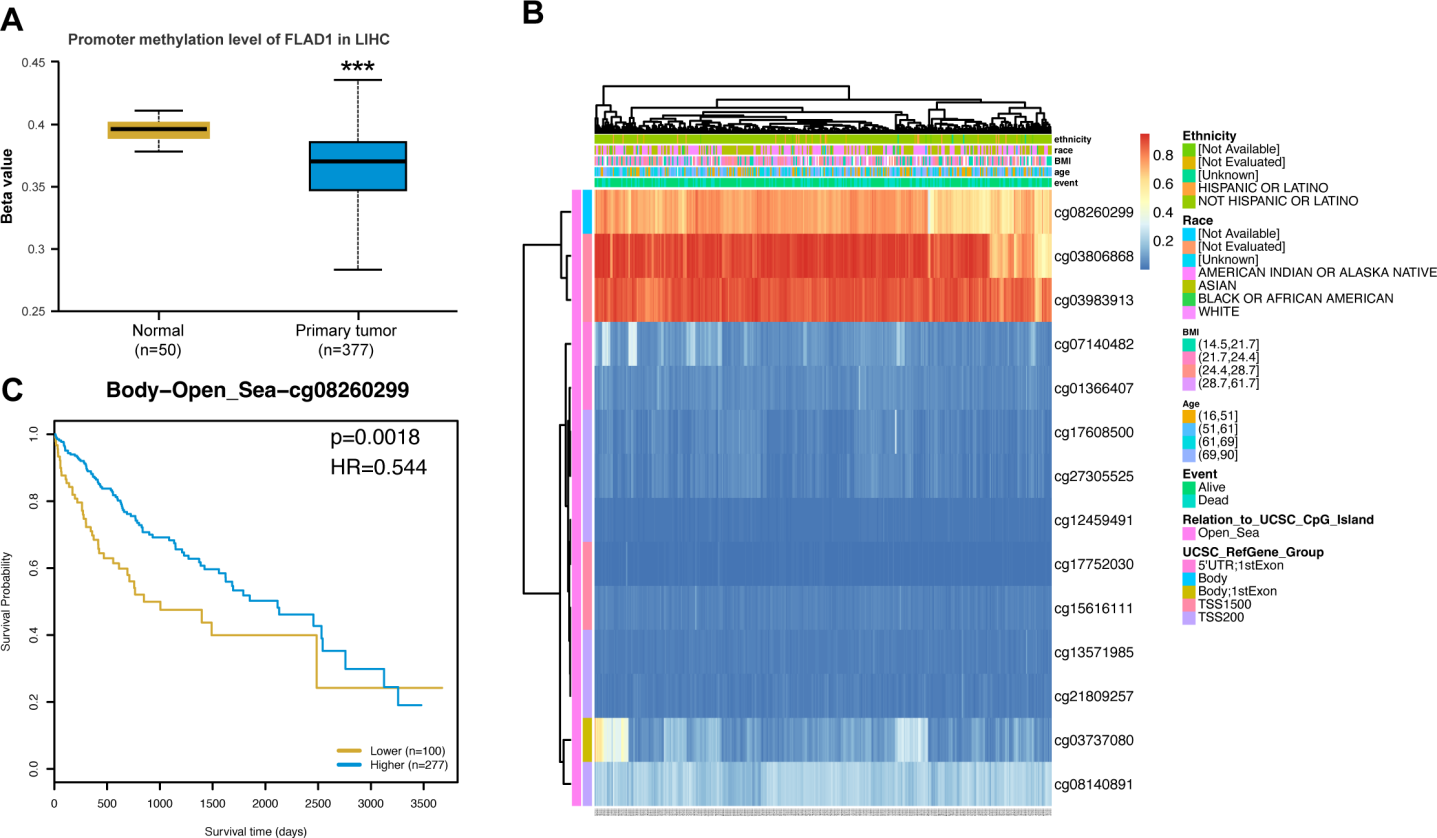
FLAD1 promoter methylation and its prognostic impact on HCC. **(A)** Lower methylation levels at the FLAD1 promoter in HCC tissues than in normal liver tissues. **(B)** Correlation between FLAD1 mRNA expression and methylation status. **(C)** Kaplan-Meier survival curves for patients with different FLAD1 methylation levels. P-values were determined using a two-tailed unpaired Student’s t test, with significance denoted as ***P < 0.001.

To investigate the mechanism underlying the up-regulation of FLAD1 in HCC, the correlation between FLAD1 expression levels and methylation status was examined. The UALCAN database revealed significantly lower DNA methylation levels at the FLAD1 promoter in HCC compared to normal liver tissues (p<0.001) (**Figure 7A**), with the majority of methylation sites in the FLAD1 DNA sequence being hypomethylated in HCC. Additionally, HCC patients with low-FLAD1 methylation exhibited poorer overall survival (OS) rates compared to those with high-FLAD1 methylation (**Figures 7B, C**).

### FLAD1 and immune infiltration

**Figure 8:**
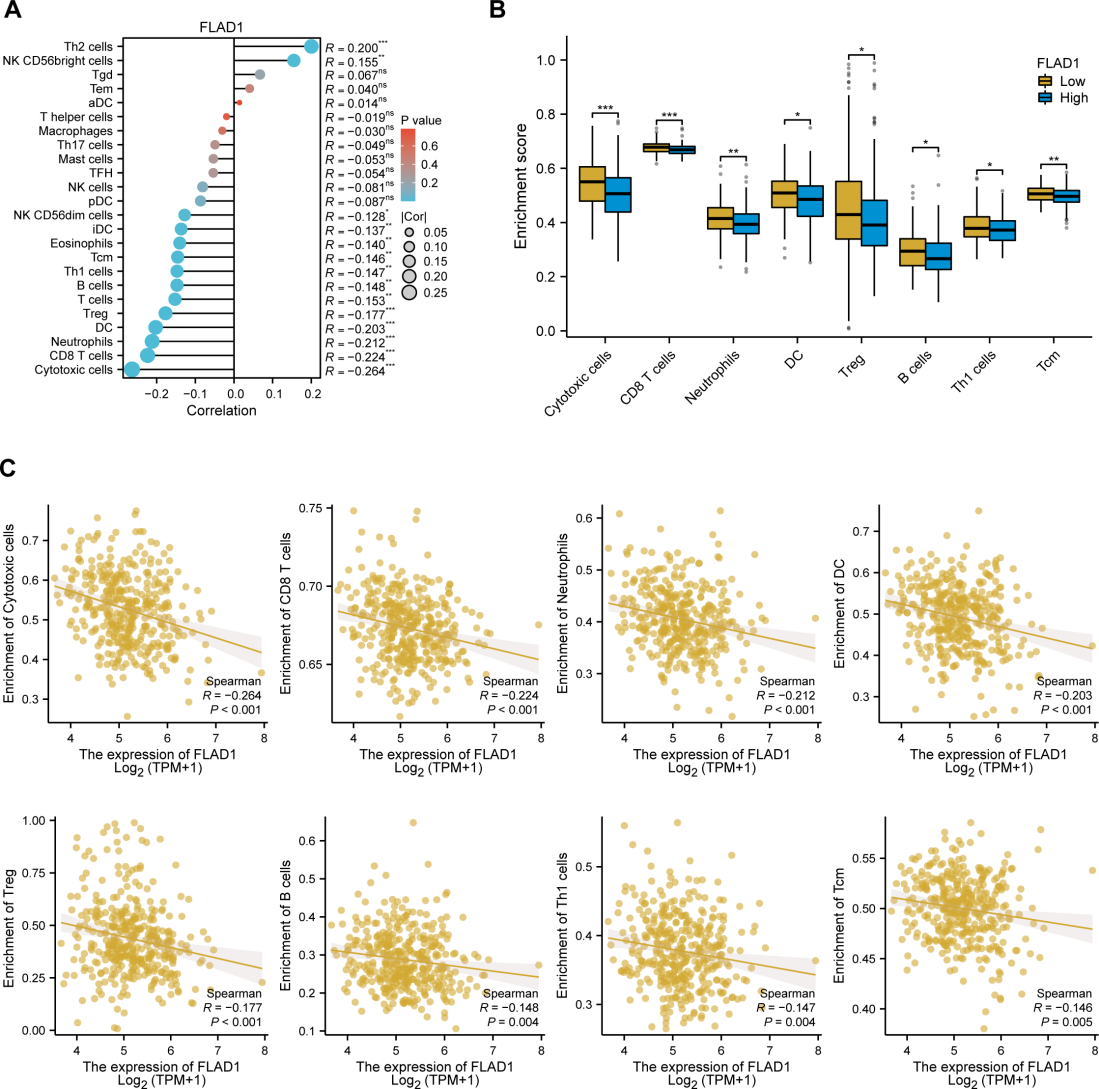
Relationship between FLAD1 expression and immune cell infiltration in HCC. **(A)** Correlations between FLAD1 expression and 24 types of immune cells. **(B)** Comparison of immune cell infiltration levels between the high- and low-FLAD1 expression groups. **(C)** Negative correlation between FLAD1 expression and the level of immune cell infiltration. P-values were calculated with two-tailed unpaired Student’s t tests, with significance denoted as *P < 0.05, **P < 0.01, and ***P < 0.001.

A negative correlation was observed between FLAD1 expression levels and the presence of various immune cells, including cytotoxic cells, CD8+ T cells, neutrophils, dendritic cells (DCs), regulatory T cells (Tregs), B cells, Th1 cells, central memory T cells (TCMs), eosinophils, immature DCs (iDCs), and NK CD56dim cells (**Figure 8A**). The analysis also showed that the group with high FLAD1 expression had significantly lower enrichment scores for these cells than did the group with low FLAD1 expression (including cytotoxic cells, CD8+ T cells, neutrophils, DCs, Tregs, B cells, Th1 cells, and TCMs), highlighting the potential immunosuppressive role of high FLAD1 expression in HCC (**Figures 8B, C**; **Supplementary Tables S9**, **10**).

### Spatial transcriptomics analysis reveals tumor capsule as an immune barrier and validates FLAD1 expression

**Figure 9:**
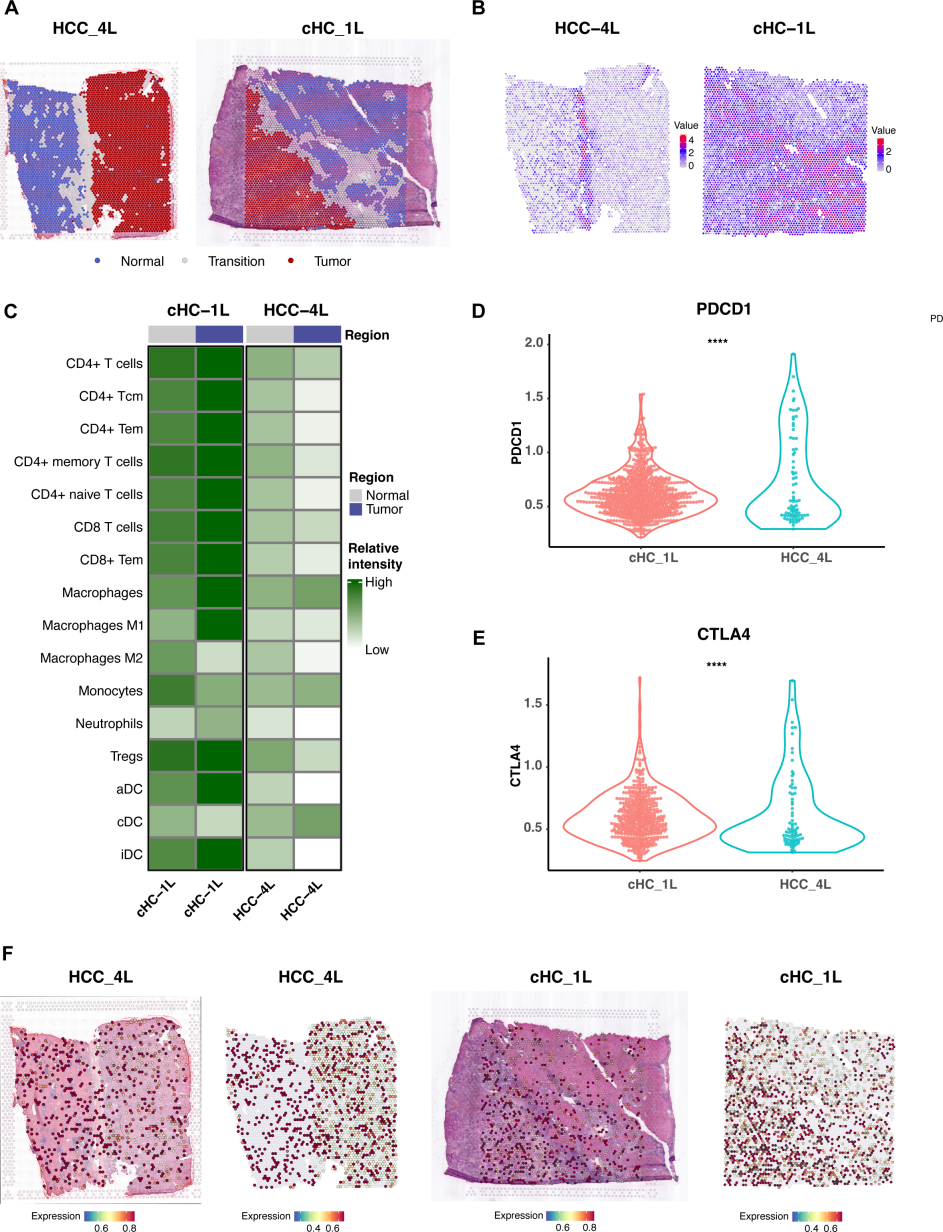
Spatial Transcriptomics Analysis Reveals Tumor Capsule as an Immune Barrier and Validates FLAD1 Expression. **(A)** Defined distribution of normal, tumor, and transition regions of the two representative HCC tissue sections used for spatial transcriptomics analysis. The left panel shows sample HCC-4L, characterized by a complete fibrous capsule (CC group). The right panel shows sample cHC-1L, which has a discontinuous or absent capsule (NC group). **(B)** Spatial feature plot illustrating the expression of the fibroblast marker COL1A1 in the HCC-4L sample. High expression is concentrated in the fibrous capsule region, delineating the tumor boundary and highlighting the dense stromal structure. **(C)** Heatmap representation of immune cell population scores in the tumor and adjacent normal regions of the CC (HCC-4L) and NC (cHC-1L) samples. **(D, E)** Comparative analysis of T-cell exhaustion markers (PDCD1 and CTLA4) in the tumor regions of the CC (HCC-4L) and NC (cHC-1L) samples. The expression levels of both markers are significantly higher in the immune-infiltrated NC sample, suggesting a state of immune dysfunction. Statistical significance was determined by the Wilcoxon test (****P < 0.0001). **(F)** Spatial feature plots validating the expression of the core diagnostic gene FLAD1 in both HCC samples.

To understand how the physical structure of HCC influences the tumor microenvironment (TME) and to spatially validate our findings, we analyzed spatial transcriptomics data from two distinct HCC samples: one with a complete capsule (CC group, HCC-4L) and another without a complete capsule (NC group, cHC-1L) (**Figure 9A**).

Our analysis revealed that the intact fibrous capsule in the HCC-4L sample was associated with a unique immune-exempt microenvironment. This was evidenced by the significantly high expression of fibroblast markers, such as COL1A1, within the capsular region (**Figure 9B**). Critically, immune cell population scores, including for T-cells and B-cells, were significantly lower within the tumor region of the CC sample compared to the adjacent normal tissue, suggesting the capsule may function as a physical barrier to immune infiltration (**Figure 9C**). In contrast, the NC sample (cHC-1L) displayed a more robust immune-infiltrated phenotype, but with signs of immune dysfunction; the expression levels of T-cell exhaustion markers like PDCD1 and CTLA4 were significantly higher than in the CC sample (P < 0.0001) (**Figures 9D, E**). Furthermore, we utilized this spatial data to validate the expression of our core diagnostic gene. Spatial feature plots confirmed that FLAD1 was expressed within the tumor regions of both the encapsulated and non-encapsulated samples (**Figure 9F**).

### Development and validation of a nomogram utilizing independent factors

**Figure 10:**
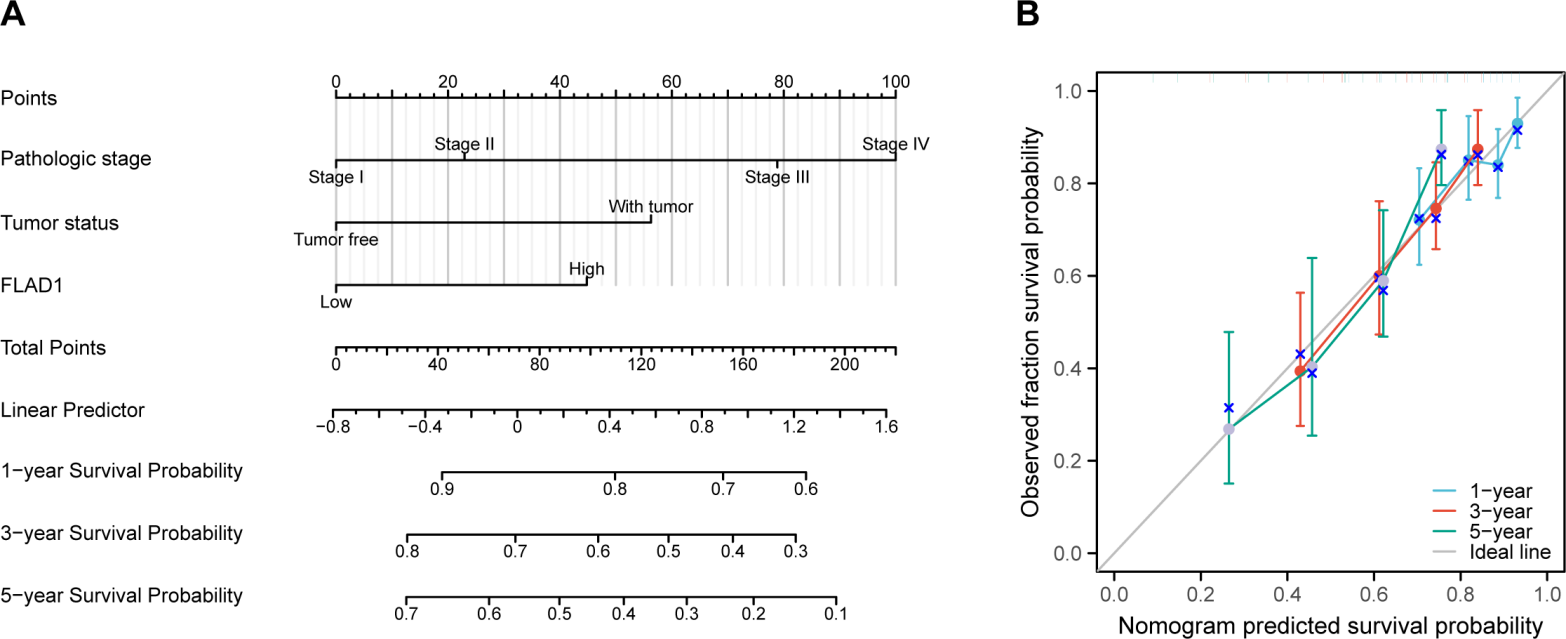
Nomogram and calibration curves for predicting OS rates in HCC patients. **(A)** The nomogram displays predictions of OS at one, three, and five years. **(B)** Calibration curves used to assess the accuracy of survival predictions at these time intervals.

A nomogram was created to forecast the prognosis of HCC patients by integrating various independent prognostic factors. The nomogram indicated that a higher total point score, representing a greater presence of adverse factors, corresponded with a worsened prognosis (**Figure 10A**). Calibration curves were used to evaluate the precision and dependability of the nomogram’s predictive efficacy (**Figure 10B**). These results confirmed that FLAD1 plays a critical role as an independent prognostic factor for HCC.

### In-house cohort validation of FLAD1 expression in HCC

**Figure 11:**
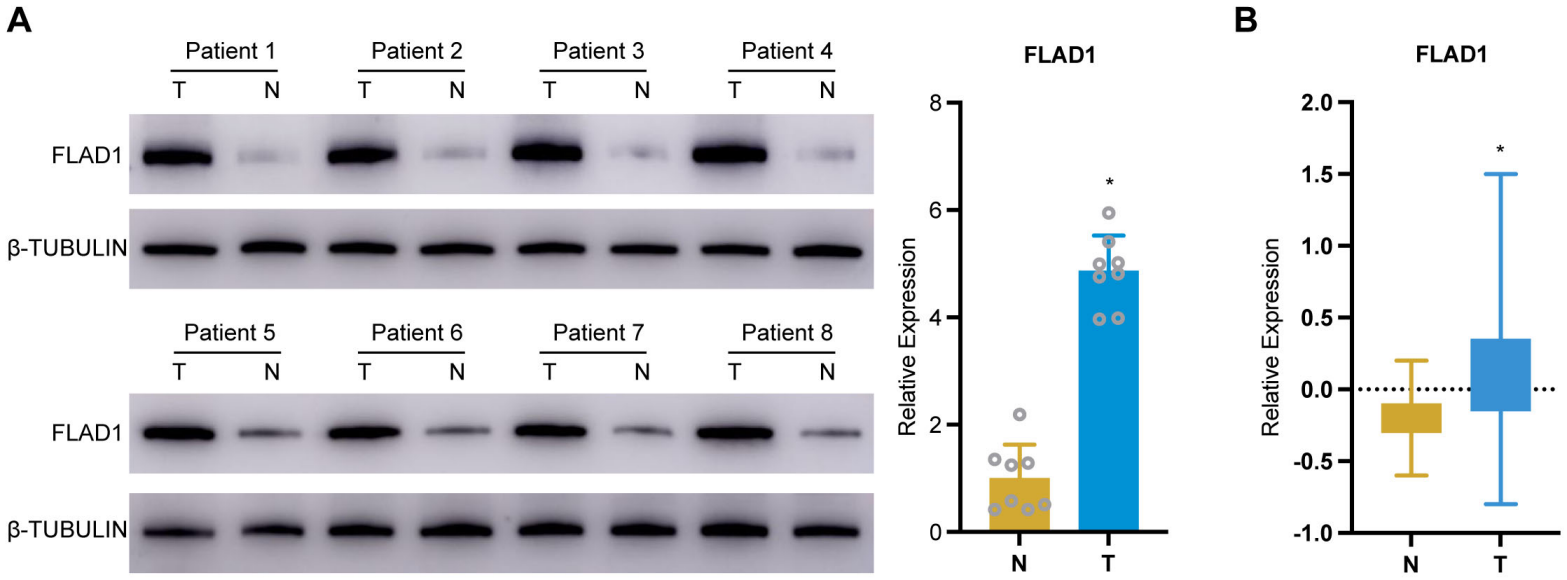
Validation of FLAD1 expression in HCC tissues. **(A)** Western blot analysis confirms increased FLAD1 protein expression in HCC tissues compared to adjacent normal liver tissues. **(B)** This box plot illustrates the relative protein expression levels of FLAD1 in HCC tissues (n=165) compared to normal liver tissues (n=165), using normalized Relative Protein Expression (nRPX) values derived from CPTAC mass spectrometry-based proteomic data (log2-transformed intensity values). *P < 0.05.

To validate the expression of FLAD1 in HCC, we conducted a series of analyses using our in-house cohort. Further molecular validation was achieved through Western blot analysis, which demonstrated a significant upregulation of FLAD1 protein levels in HCC tissue samples compared to adjacent normal liver tissues, confirming the enhanced expression of FLAD1 in the cancerous tissues (**Figure 11A**). This finding was corroborated by CPTAC cohort from the Human Protein Atlas (HPA) database, which also showed a significantly increase of FLAD1 expression in HCC samples (n=165) relative to healthy liver samples (n=165) (p<0.0001, **Figure 11B**). These comprehensive analyses from our in-house cohort confirm the role of FLAD1 as a critical biomarker in HCC, highlighting its potential clinical significance in the context of liver cancer diagnostics and treatment planning. Advanced imaging techniques such as computed tomography (CT) scans, including non-contrast scans, arterial phase hyperenhancement (APHE) in both conventional and thin slice modalities, as well as three-dimensional reconstructions in coronal and sagittal planes during both portal venous and parenchymal phases (**Supplementary Figure S3**), revealed distinct variations between HCC lesions and normal liver tissues. These imaging results underscore the localized changes in liver architecture associated with cancer progression.

### Structural prediction and molecular docking analysis of FLAD1

**Figure 12:**
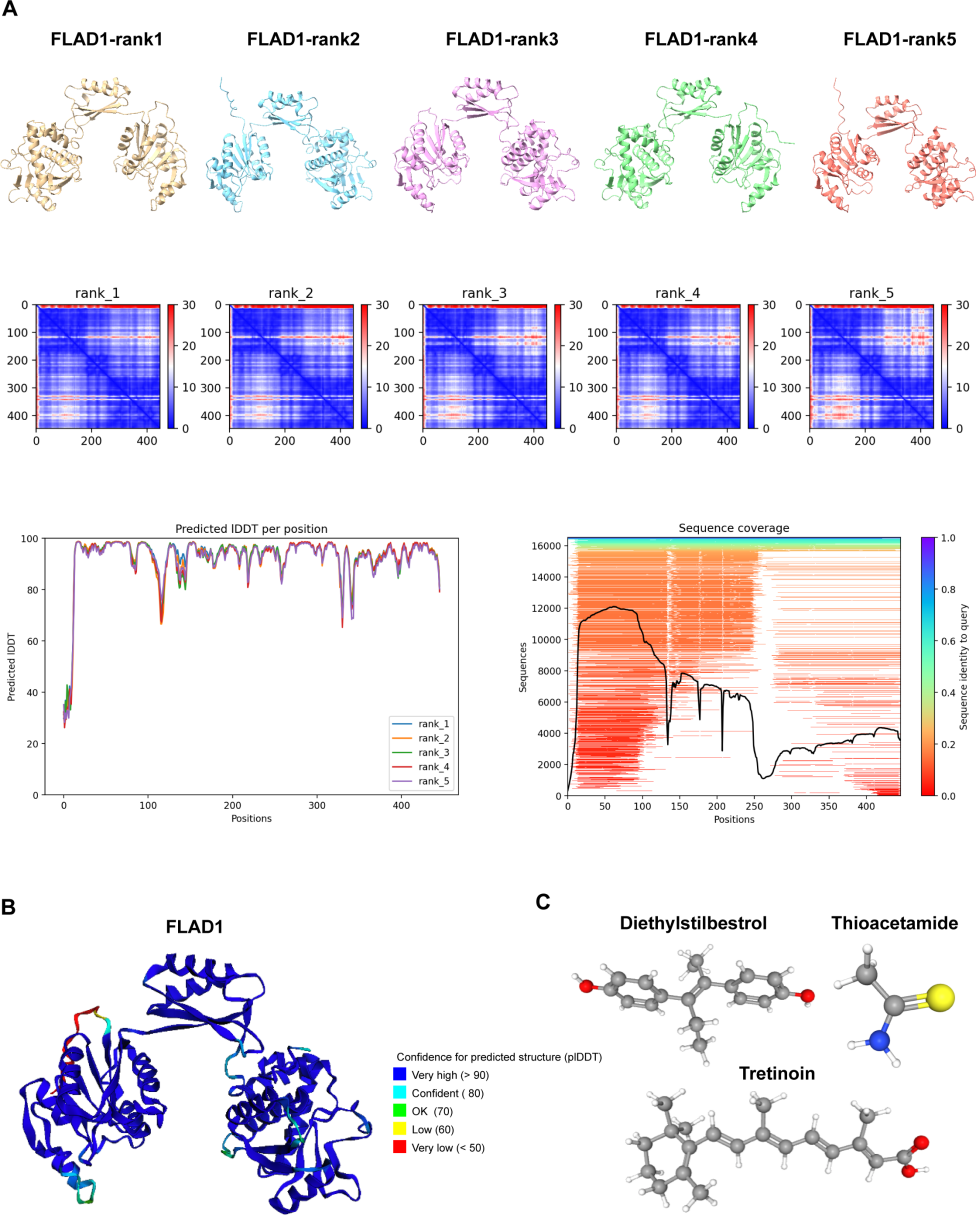
Predictive Modeling of FLAD1 Protein Structures Using AlphaFold 2. **(A)** AlphaFold 2 was utilized to predict the structures of FLAD1 under default conditions, with the models ranked by confidence. The predicted structures are displayed in various colors (yellow, blue, purple, green, orange), each corresponding to their Predicted Aligned Error (PAE), predicted Local Distance Difference Test (plDDT) values, and sequence coverage, illustrating the accuracy and reliability of the predictions. **(B)** The highest-ranked AlphaFold 2 model of FLAD1 (rank1) is depicted, showcasing the predicted folding and conformational dynamics of the protein within a cellular context. This model highlights the structural intricacies and potential functional domains of FLAD1. **(C)** Three-dimensional molecular structures of Diethylstilbestrol, Thioacetamide, and Tretinoin are illustrated, emphasizing their unique chemical configurations.

Using AlphaFold 2 technology, we accurately predicted the intricate three-dimensional structure of FLAD1, demonstrating the predictive reliability of this structural conformation (**Figures 12A, B**). The AlphaFold 2 predictions, performed under default conditions, were ranked by model confidence and are displayed in an array of colors (yellow, blue, purple, green, orange), each corresponding to their Predicted Aligned Error (PAE), predicted Local Distance Difference Test (plDDT) values, and sequence coverage.

**Figure 13:**
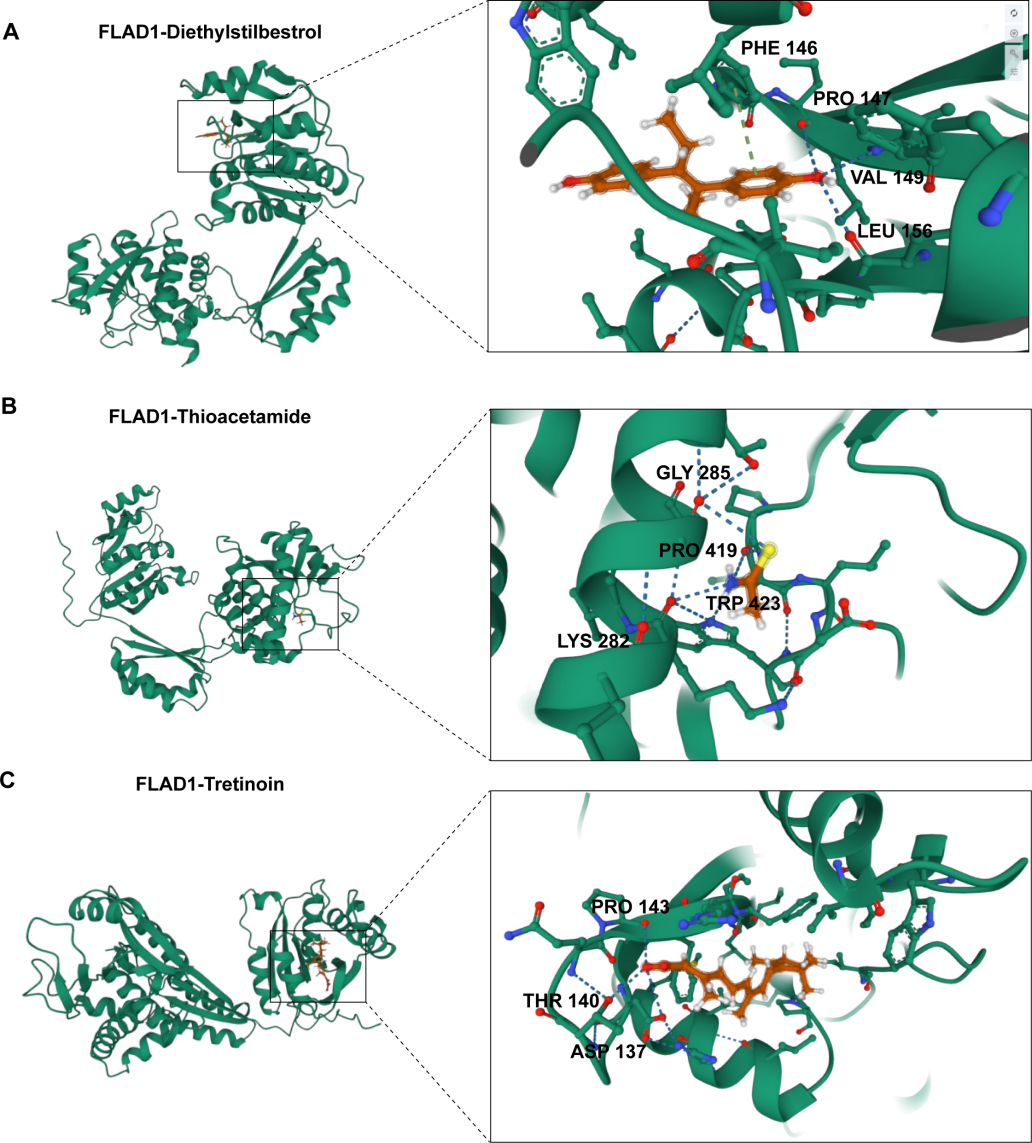
Predictive modeling of the interactions of FLAD1 with various ligands using molecular docking techniques. **(A)** Interaction analysis of Diethylstilbestrol with the FLAD1 protein through molecular docking, highlighting the binding efficiency and potential sites of interaction. **(B)** Docking simulation of Thioacetamide with FLAD1, demonstrating the molecular engagement and potential impact on FLAD1 function. **(C)** Tretinoin’s docking with FLAD1 depicted to reveal the binding patterns and interaction dynamics, which may influence the biological activity of FLAD1.

To investigate the therapeutic potential of targeting FLAD1, molecular docking studies were conducted using AutoDock Vina v.1.1.2. We assessed the interactions of FLAD1 with potential therapeutic agents—Diethylstilbestrol, Thioacetamide, and Tretinoin (**Figure 12C**). The resulting analyses indicated binding energies of -6.959 kcal/mol for FLAD1-Diethylstilbestrol, -2.88 kcal/mol for FLAD1-Thioacetamide, and -8.959 kcal/mol for FLAD1-Tretinoin (**Figures 13A–C**). The strong binding affinities observed with Diethylstilbestrol and Tretinoin, in particular, underscore their significant therapeutic promise in targeting FLAD1 for the treatment of HCC.

## Discussion

Hepatocellular carcinoma (HCC) often remains asymptomatic until it reaches advanced stages, leading to late diagnosis and limited treatment options (59). The multifactorial etiology of HCC underscores the complexity of its pathogenesis and the challenge in developing effective therapies (3). The burden of HCC on healthcare systems and the quality of life of affected individuals is substantial, necessitating research into novel diagnostic and therapeutic approaches.

Recent advancements in the integration of molecular profiling with clinical phenotypes have shown promise in enhancing the understanding and management of HCC. The exploration of mRNA expression patterns and their association with clinical outcomes can lead to the identification of novel biomarkers and therapeutic targets, potentially transforming the landscape of HCC treatment (3). Furthermore, single-cell RNA sequencing offers an unprecedented resolution to dissect the heterogeneity within tumor microenvironments, which contributes to personalized medicine approaches that could significantly improve patient care (60). The application of machine learning algorithms in predicting disease outcomes based on gene signatures further exemplifies the potential of bioinformatics in revolutionizing HCC prognosis and treatment selection (61).

The study of gene expression profiles and molecular pathways in HCC could revolutionize the management of this disease. Flavin adenine nucleotide synthetase 1 (FLAD1), which was initially not recognized as encoding a mitochondrial protein, has been identified as a gene that produces cytoplasmic and mitochondrial transcripts, making it a potential candidate gene (11). Variants in FLAD1 have been associated with multiple acyl-CoA dehydrogenase deficiencies and combined respiratory chain deficiencies, indicating a broader impact on the flavinylation of mitochondrial proteins (62). Recent research has highlighted FLAD1 variants as a cause of riboflavin-induced disorders, emphasizing their role in mitochondrial function. Furthermore, FLAD1 deficiency leads to lipid storage myopathy, demonstrating its involvement in mitochondrial dysfunction (63). FLAD1 has recently emerged as a potential player in cancer progression (64–66). Understanding the differential expression of FLAD1 and its association with disease phenotypes could unveil the identification of novel biomarkers. The exploration of the role of FLAD1 in immune cell infiltration and DNA methylation patterns offers a promising avenue for the development of personalized medicine strategies, potentially improving patient outcomes and survival rates.

The notable upregulation of FLAD1 in HCC tissues relative to normal and adjacent nontumor tissues suggests its potential involvement in tumorigenesis and progression. This is further supported by its association with higher pathological stage, elevated alpha-fetoprotein (AFP) levels, and poor prognosis, indicating FLAD1’s potential as an HCC biomarker and could be targeted for therapeutic intervention.

The discovery of 1131 differentially expressed genes (DEGs) related to FLAD1 expression in HCC, 692 of which were upregulated and 439 of which were downregulated, underscores the influence of this gene on cellular processes. Enrichment analyses revealed the involvement of these DEGs in vital biological pathways like copper ion detoxification, stress response, and neuroactive ligand-receptor interaction, which are critical for maintaining cellular homeostasis and could be implicated in the pathophysiology of HCC.

Furthermore, functional network analysis using GeneMANIA highlighted the interaction of FLAD1 with genes implicated in vital metabolic pathways, including vitamin metabolism and hexose metabolism. Crucially, our analysis confirmed that this entire 20-gene functional network is differentially expressed in high-FLAD1 HCC, indicating that these gene-gene interactions are not only functionally linked but also empirically altered in the tumor context. This finding provides strong evidence that the FLAD1-centric network is an active, transcriptionally dysregulated module in HCC. These interactions are pivotal for the altered metabolic demands of cancer cells, a phenomenon known as the Warburg effect, where cancer cells favor glycolysis over oxidative phosphorylation, even when oxygen is available (67–69). The dysregulation of this FLAD1-associated metabolic network reinforces its role in fueling the bioenergetic needs for tumor proliferation and survival.

Furthermore, the negative correlation between FLAD1 expression and DNA methylation status, particularly in the promoter region of HCC tissues, suggests epigenetic regulation as a potential mechanism for FLAD1 overexpression. Reduced methylation levels could lead to the transcriptional activation of FLAD1, contributing to the oncogenic phenotype observed in HCC. This finding opens new options for understanding the epigenetic landscape of HCC and the role of DNA methylation in regulating oncogenes and tumor suppressor genes.

In HCC, immune cell infiltration has emerged as a significant prognostic indicator, with various immune cell types exerting either tumor-promoting or tumor-suppressing effects (70, 71). Our study highlights the significant correlation between FLAD1 expression and immune cell infiltration levels, particularly the negative association between FLAD1 expression and the presence of key effector cells such as CD8+ T cells and dendritic cells. These discoveries imply that FLAD1 may modulate the tumor microenvironment, potentially creating a more immunosuppressive environment that facilitates tumor growth and progression.

Our spatial transcriptomics analysis provides a potential physical basis for this observation, demonstrating that the fibrous capsule in some HCC tumors acts as a barrier, effectively creating an immune-exempt niche by preventing immune cell entry. This finding suggests that for encapsulated tumors, the mechanism of immune evasion may be structural as well as biological.

The diminished presence of CD8+ T cells in the context of high FLAD1 expression is particularly noteworthy, given their established role in mediating antitumor immune responses (72). Dendritic cells, on the other hand, are crucial for antigen presentation and the initiation of adaptive immune responses (73). A reduction in their numbers could lead to impaired immune surveillance and a less effective immune response against HCC. The association of FLAD1 with these immune cells underscores the potential of FLAD1 not only as a prognostic indicator for HCC but also as therapeutic intervention target aimed at modulating the immune response.

Furthermore, our analysis suggested that the prognostic value of FLAD1 may be partially attributable to its influence on the immune landscape of HCC. By integrating gene expression data with immune cell infiltration levels, we provide a more comprehensive understanding of the tumor-immune interface in HCC. This integrative approach could shed light to the development of combination therapies that target both molecular aberrations in cancer cells and associated immune evasion mechanisms (74, 75).

Reflecting on the limitations of this study, it is important to acknowledge certain constraints that may affect the interpretability of our findings. While our bioinformatic findings are based on robust sample sizes, the protein-level validation is preliminary and requires confirmation in a larger clinical cohort. Additionally, the lack of clinical validation analysis is another limitation, as it is crucial to confirm the relevance of our findings in a clinical setting. These limitations should be carefully considered when interpreting the study’s outcomes.

In this study, we utilized scRNA-seq for detailed analysis, developed a machine-learning model to predict mitochondrial-related gene patterns, and conducted molecular experiments to elucidate the role of FLAD1 in HCC. Our findings reveal unique expression patterns of mitochondrial-related genes and the development of a highly accurate predictive model. Moreover, significant changes in FLAD1 expression, along with its associations with immune cell infiltration and DNA methylation, highlight its dual potential as both a diagnostic marker and therapeutic target in HCC. The application of scRNA-seq and machine learning has not only deepened our understanding of the molecular intricacies of HCC but also opened new options for targeted therapeutic strategies.

## Data Availability

The original contributions presented in the study are included in the article/**Supplementary Material**. Further inquiries can be directed to the corresponding authors.

## Supplementary material

Supplementary Figure 1 Validation of machine learning models for predicting mitochondrial gene expression in HCC by external ICGC cohort. AUC scores for 92 machine learning algorithm combinations demonstrating model performance.

Supplementary Figure 2 Differential expression of hub MitRGs in HCC. **(A)** The mRNA levels of central MitRGs in normal and HCC tissues, comparing data from TCGA and GTEx databases to showcase expression alterations. **(B)** Comparative analysis of MitRG expression in HCC tumors versus adjacent non-tumor liver tissues from TCGA, highlighting the genes with significant differential expression. **(C)** ROC curve of MitRG. Statistical significance is indicated with P-values derived from unpaired **(A)** and paired t-tests **(B)**. *P < 0.05, **P < 0.01 and ***P < 0.001.

Supplementary Figure 3 Computed Tomography scans of HCC patients. Images include non-contrast CT, arterial phase hyperenhancement (APHE) in conventional, thin slice, and thin slice coronal three-dimensional reconstruction views (3D), along with portal venous phase and parenchymal phase in conventional, thin slice, thin slice sagittal 3D, and thin slice coronal 3D reconstructions.

Supplementary Table 1 Clinical Characteristics of the In-House Validation Cohort.

Supplementary Table 2 Differentially expressed genes between metastatic and primary hepatocellular carcinoma (HCC).

Supplementary Table 3 The mRNA expression levels of hub Mitochondrial-related gene (MitRG) in normal liver tissues from healthy individuals and HCC tumor tissues from the TCGA and GTEx databases.

Supplementary Table 4 Comparison of hub MitRG expression between HCC tumor tissues and paired cancer-adjacent tissues from TCGA data.

Supplementary Table 5 The upregulated and downregulated differentially expressed genes between HCC tissues and paired cancer-adjacent tissues from TCGA data.

Supplementary Table 6 The top ten DEGs positively correlated with FLAD1.

Supplementary Table 7 Gene Ontology (GO) and Kyoto Encyclopedia of Genes and Genomes (KEGG) analyses of DEGs.

Supplementary Table 8 Gene Set Enrichment Analysis (GSEA) of DEGs.

Supplementary Table 9 Comparison of immune cell infiltration levels between the high- and low-FLAD1 expression groups.

Supplementary Table 10 There was a negative correlation between FLAD1 expression and the level of immune cell infiltration.
