# Supplementary figures and images for: Integrative single-cell and spatial transcriptomics analysis reveals FLAD1 as a regulator of the immune microenvironment in hepatocellular carcinoma
Source: Front Immunol. 2025 Oct 29;16:1680101. doi: 10.3389/fimmu.2025.1680101 (PMC12605016; doi:10.3389/fimmu.2025.1680101)

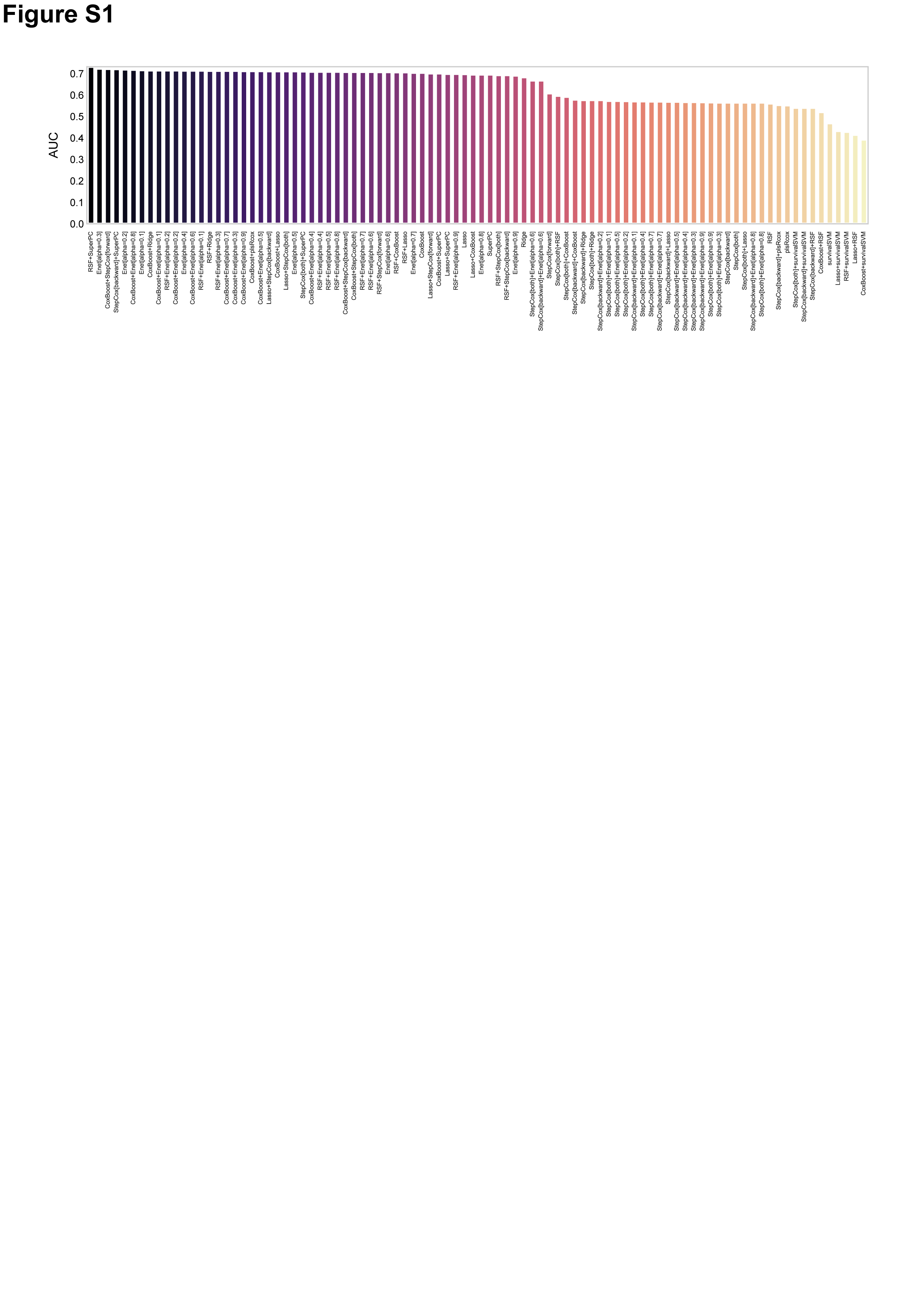

Supplement: Supplementary Figure 1 — Validation of machine learning models for predicting mitochondrial gene expression in HCC by external ICGC cohort. AUC scores for 92 machine learning algorithm combinations demonstrating model performance. [file Image1.tif]

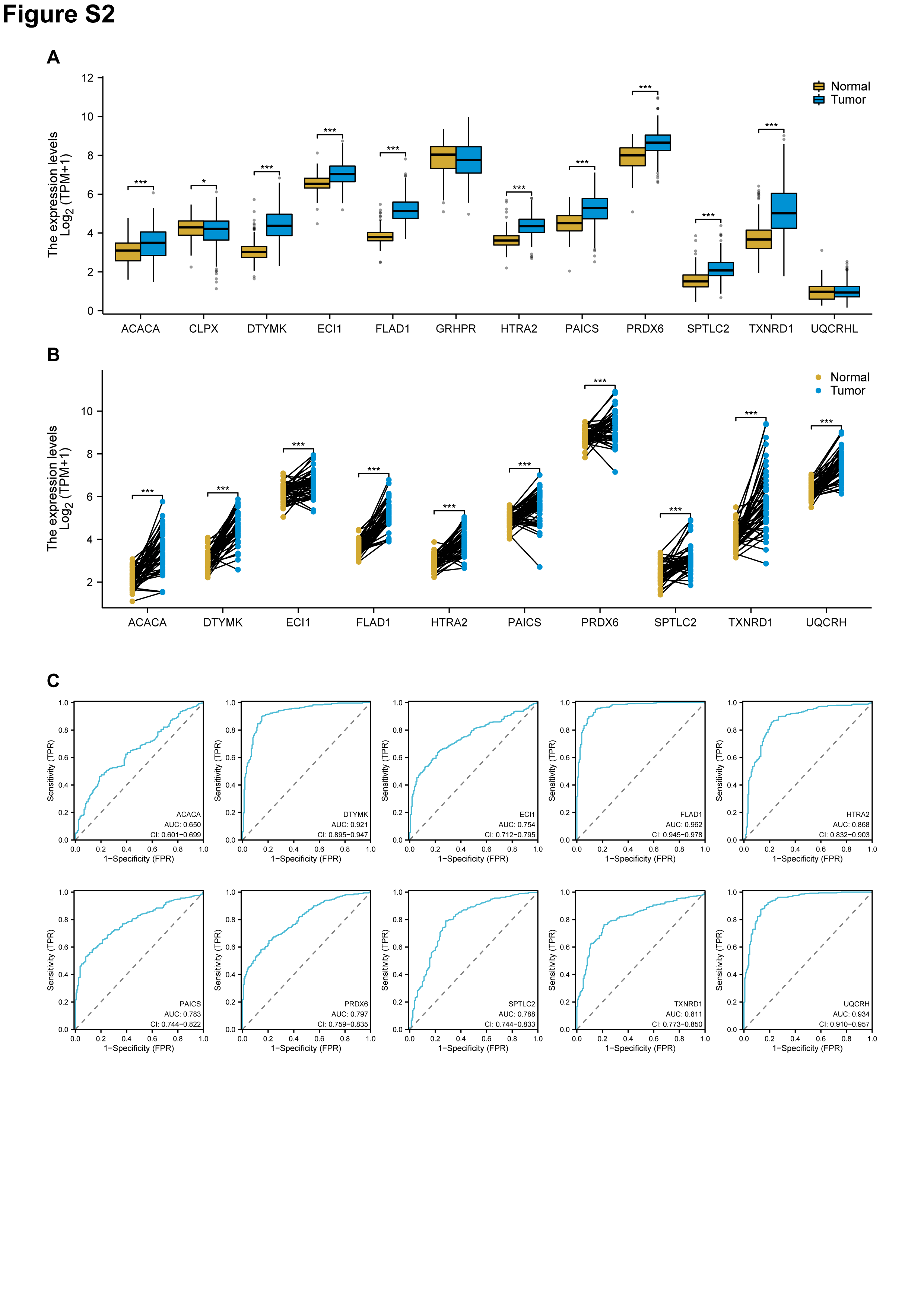

Supplement: Supplementary Figure 2 — Differential expression of hub MitRGs in HCC. (A) The mRNA levels of central MitRGs in normal and HCC tissues, comparing data from TCGA and GTEx databases to showcase expression alterations. (B) Comparative analysis of MitRG expression in HCC tumors versus adjacent non-tumor liver tissues from TCGA, highlighting the genes with significant differential expression. (C) ROC curve of MitRG. Statistical significance is indicated with P-values derived from unpaired (A) and paired t-tests (B). *P < 0.05, **P < 0.01 and ***P < 0.001. [file Image2.tif]

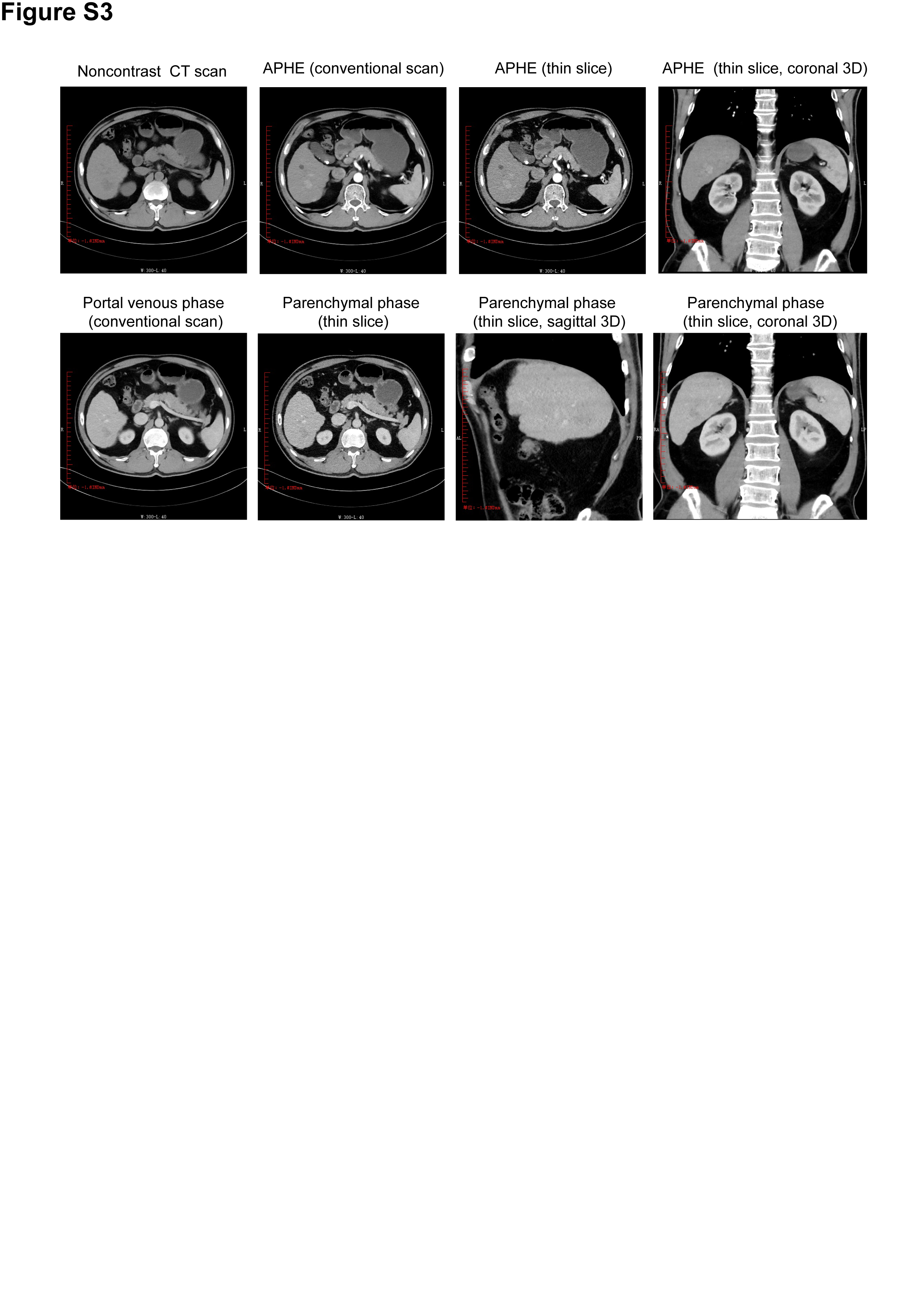

Supplement: Supplementary Figure 3 — Computed Tomography scans of HCC patients. Images include non-contrast CT, arterial phase hyperenhancement (APHE) in conventional, thin slice, and thin slice coronal three-dimensional reconstruction views (3D), along with portal venous phase and parenchymal phase in conventional, thin slice, thin slice sagittal 3D, and thin slice coronal 3D reconstructions. [file Image3.tif]
